# Nigrostriatal dynein changes in A53T alpha-synuclein transgenic mice

**DOI:** 10.12688/f1000research.3507.1

**Published:** 2014-03-11

**Authors:** Yan Liu, Yu-He Yuan, Jian-Dong Sun, Jing Li, Zhi-Peng Li, Nai-Hong Chen

**Affiliations:** 1 State Key Laboratory of Bioactive Substances and Functions of Natural Medicines, Department of Pharmacology, Institute of Materia Medica, Chinese Academy of Medical Sciences and Peking Union Medical College, Beijing, 100050, China; 2 Tianjin University of Traditional Chinese Medicine, Tianjin, 300193, China

## Abstract

The accumulation of misfolded a-synuclein is mechanistically linked to neurodegeneration in Parkinson’s disease (PD) and other alpha-synucleinopathies. However, how alpha-synuclein causes neurodegeneration is unresolved. Several studies have supported the involvement of dynein, the major motor for retrograde axonal transport in alpha-synuclein-dependent neurodegeneration, especially in the nigrostriatal system. Therefore, we examined the nigrostriatal dyneins in transgenic mice that overexpress human A53T alpha-synuclein and recapitulate key features of a PD-like neuronal synucleinopathy. Age-matched nontransgenic littermates were used as controls. The results demonstrated that the protein level of dynein was decreased in the striatum, whereas it was elevated in the substantia nigra. Double immunostaining results revealed that the reduction in dynein level was associated with aggregation of A53T a-synuclein in the striatum. Furthermore, we performed a quantitative analysis of motor behaviors in A53T alpha-synuclein transgenic mice and controls using a modified open field test. We demonstrated that the protein level of dynein in the striatum was significantly correlated with the motor behaviors. Together, our data indicate that dynein changes in the nigrostriatal system of A53T alpha-synuclein transgenic mice may contribute to their severe movement disorder.

## Introduction

α-Synuclein abnormalities are mechanistically linked to the pathogenesis of Parkinson’s disease (PD) and other α-synucleinopathies. α-Synuclein is the major component of Lewy bodies, the neuropathological hallmarks of PD ^1, 2^. Duplication, triplication or mutations in the α-synuclein gene cause some forms of familial PD ^3, 4^. However, the mechanism whereby α-synuclein promotes neurodegeneration remains unclear.

Dyneins are minus end-directed microtubule motors that move cargoes such as mitochondria, organelles and proteins from the distal ends of axons toward neuronal cell bodies ^5, 6^. Dynein is the major motor of retrograde axonal transport, and it is also the molecular motor responsible for the transport of misfolded proteins to be degraded. Therefore, it is crucially involved in the appearance and clearance of protein aggregates ^7^. Several studies have supported the involvement of dynein in the neurodegeneration associated with PD. First, recent studies suggest that axonal transport disruption may be causal to disease progression in PD ^8, 9^. Alterations in axonal transport motor proteins have been observed in postmortem patient brain samples ^9^ and treatment with 1-methyl-4-phenyl-1,2,3,6-tetrahydropyridine (MPTP), a PD-relevant injury, leads to defective axonal transport, including increased dynein-dependent transport ^10^. Second, a number of dynein-dependent processes, including autophagy or clearance of aggregation-prone proteins, are found to be defective in PD ^11^. In all cases, defects in axonal transport and autophagy occurring in PD indicate that dynein may be a central factor in PD pathology. Interestingly, experimental evidence suggests that α-synuclein mutations (A53T, A30P) might lead to axonal transport defects both *in vivo* and *in vitro* ^8, 9, 12^. Therefore, we hypothesized that the neurodegeneration observed in the A53T mutant human α-synuclein transgenic (Tg) mouse model of α-synucleinopathy was associated with alterations of dynein.

In this study, we first evaluated the motor function of A53T human α-synuclein Tg mice and age-matched non-transgenic (nTg) littermates using a modified open field test. Unlike previous studies that only provided behavioral descriptions, in this study, we quantified behavior. We then examined the expression of dynein in the striatum and substantia nigra (SN), as dynein defects have been mostly studied in the nigrostriatal system in previous research ^9^. To evaluate whether changes of dynein were related to α-synuclein aggregation, double immunostaining for α-synuclein and dynein was performed. In addition, we analyzed the correlation between the motor behaviors and the protein level of dynein in the striatum. Our findings reveal that dynein changes in the nigrostriatal system of A53T α-synuclein Tg mice may contribute to their dramatic motor phenotype.

## Materials and methods

### Animals

Animal experiments were conducted in accordance with the principles and procedures of the US National Institutes of Health Guide for the Care and Use of Laboratory Animals. All protocols were approved by the Institutional Animal Care and Use Committee of Peking Union Medical College and Chinese Academy of Medical Sciences.

The generation of Tg mice expressing high levels of mutant A53T α-synuclein under the control of the mouse prion protein (PrP) promoter has been described ^13^. Mice expressing A53T α-synuclein (line M83), but not mice expressing wild type α-synuclein, develop adult-onset progressive motor deficits ^13^. According to the first report of M83 mice ^13^, about 50%–70% of mice at 10–14 months of age develop the motor phenotype. Original mice were obtained from The Jackson Laboratory. We purchased the mice from Model Animal research Center of Nanjing University. In all experiments 12 male M83 mice aged between 10–14 months were used. Eight control mice were age-matched, male, nTg littermates. No statistical method was used to pre-determine sample size; however, the sample size per experiment was based on our previous successful experiments and publications. Mice were raised on a 12-h light/dark cycle, with food and water available ad libitum and were housed in groups of four per cage.

### Modified open field test

**Figure 1.**
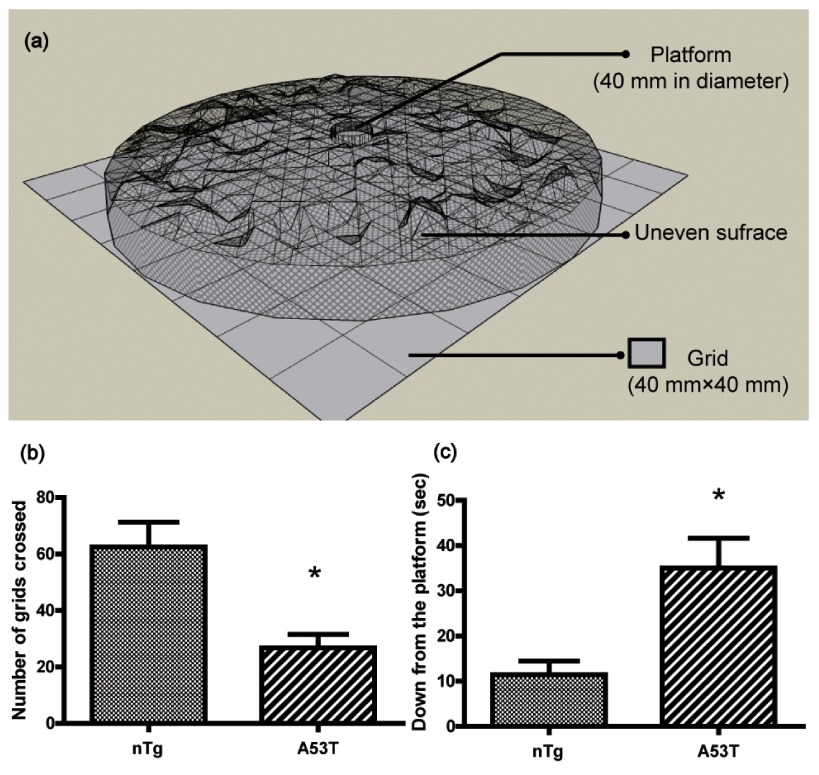
Motor behaviors of A53T α-synuclein Tg mice (10–14 months old) and age-matched nTg littermates were evaluated a using modified open field test. ( **a**) 3-D model for modified open-field test apparatus. The data (mean ± SEM) show the number of grids crossed ( **b**) and the time to get down from the platform ( **c**) in a 6 min period in an open field (nTg, n = 8; A53T, n = 12). ^*^ *p*<0.05 compared to nTg.

The procedure was modified from the protocol previously described ^14^. The apparatus consisted of a rectangular area of 32 × 32 cm which was divided into 64 squares of 4 × 4 cm. An uneven surface (32 cm in diameter) made of mesh wire (200 mesh) was raised by a ring in this area. A platform (4 cm in diameter) was placed in the middle of the uneven surface ( Figure 1a). The day before the test, the mice were given two trials. During the test, the animals were placed on the platform and their activities were assessed during the subsequent 6 min period. The performance of mice was video recorded. Horizontal locomotion (number of grids crossed) and latency (time to get down from the platform) were analyzed thereafter.

### Western blotting

Total protein extracts from striatum and ventral midbrain were prepared and western blot analyses were performed as described previously ^15, 16^. Primary antibodies were as follows: mouse anti-α-synuclein (Syn204) monoclonal antibody (Cell Signaling Technology, #2647), mouse anti-dynein monoclonal antibody (Millipore, #MAB1618), rabbit anti-dynein intermediate chain polyclonal antibody (Abcam, #ab81507). Horseradish peroxidase-conjugated secondary antibody (KPL, 1:5000) and enhanced chemiluminescence solution (Applygen Technologies Inc) were used for detection. Bands were quantified using Gel-Pro Analyzer software (Media Cybernetics).

### Immunohistochemistry and double-labeling immunofluorescence

Mice were anesthetized with 10% chloral hydrate and then perfused through the heart for 3 min with PBS followed by 4% paraformaldehyde (50–100 ml). The brains were then removed, postfixed with 4% paraformaldehyde for 4 hours, embedded in paraffin as described by OpenWetWare ( http://openwetware.org/wiki/Paraffin_embedding_and_sectioning), and cut into 3μm serial sections using Leica tissue slicer (RM2235). Stainings were all performed according to our protocol for immunostaining ^16, 17^. Immunohistochemistry was performed with mouse anti-dynein antibody (Millipore, 1:100). Double immunofluorescence was performed by using rabbit anti-dynein antibody (Abcam, #ab121209, 1:50) and mouse anti-α-synuclein antibody (Cell Signaling Technology, #2647, 1:50). Immunostaining was visualized by 3,3-diaminobenzidine or by fluorescein isothiocyanate and Alexa 546 (Invitrogen, 1:100) and was examined by either regular light or laser-scanning confocal microscope (Zeiss, Germany).

### Image analyses

Image analyses were performed according to previously published procedures ^9, 18, 19^. Briefly, a 1 × 0.5 mm ^2^ contour was placed over the striatum at low magnification (×4 objective) and the optical density of dynein-immunoreactivity (dynein-ir) within the contour were measured under high magnification (×40 objective). Each subfield of the substantia nigra pars compacta (SNpc) was manually outlined at low magnification (×4 objective). 50% of the fields were randomly selected. Then, at high magnification (×40 objective), each selected field was retrieved automatically. Optical density measurements were performed on individual tyrosine hydroxylase-positive neurons which had been stained on adjacent sections. The optical density of dynein-ir in the striatum and SNpc was measured for six sections per animal (1 out of every 20 serial sections) using Image-Pro Plus software (Media Cybernetics).

### Statistical analysis

The results are expressed as the mean ± SEM. Statistical significances were determined by two-tailed Student’s t-test. Pearson’s correlation test was used to analyze the correlation between motor behaviors and dynein protein level. The level of statistical significance was set at *p*<0.05. All analyses were conducted by the statistical software package SPSS 13.0 for Windows.

## Results

### Modified open field test

Consistent with a previous report ^13^, a few homozygous mice expressing A53T α-synuclein developed a progressively severe motor phenotype at 8 months of age. No behavioral tests were performed on the transgenic mice before 8 months of age. In the present study, we used a modified open field test ^14^ to quantitatively evaluate the motor behaviors of the mice ( Figure 1a). A53T human α-synuclein Tg mice showed a 60% decrease in the number of total grids crossed compared to nTg mice ( *p*>0.05, Figure 1b), and A53T mice took much longer (3-fold) to get down from the platform than nTg mice ( *p*<0.05, Figure 1c), indicating decreased motor function.

### Protein level of dynein

**Figure 2.**
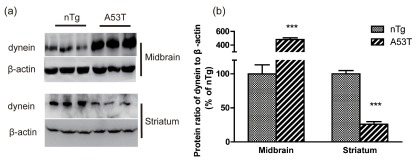
Western blot analyses of dynein in the striatum and midbrain. ( **a**) Representative western blot images of dynein and β-actin in the striatum and midbrain are shown, and ( **b**) the results were quantified (mean ± SEM) and normalized by the averaged value of nTg group. nTg, n = 5; A53T, n = 8; ^***^ *p*<0.001 compared to nTg.

Western blotting was used to detect dynein protein expression in the striatum and ventral midbrain. The levels of dynein were significantly decreased in the striatum of A53T human α-synuclein Tg mice compared to nTg mice ( *p*<0.001, Figure 2a, b). In contrast to the results in the striatum, the expression levels of dynein were upregulated in the ventral midbrain ( *p*<0.001, Figure 2a, b).

**Figure 3.**
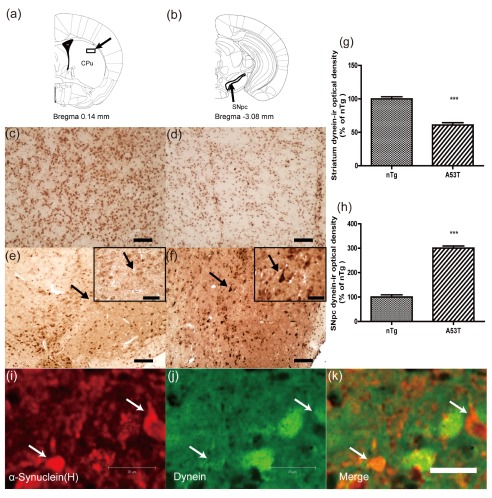
Immunohistochemical analyses of dynein in the striatum and substantia nigra pars compacta (SNpc). Optical density of dynein-immunoreactivity was quantified in the striatum and SNpc as shown in ( **a**) and ( **b**) ^26^. The photomicrographs show the distribution of immunoreactivity for dynein in the striatum ( **c**– **d**) and SNpc ( **e**– **f**). Sections were prepared from nTg mice ( **c**, **e**) and A53T α-synuclein Tg mice ( **d**, **f**). The immunoreactivity for dynein was decreased in the striatum of A53T α-synuclein Tg mice but increased in the SNpc. Insets in ( **e**) and ( **f**) are high-magnification images of the region indicated by arrows. Abundant dynein accumulation in neuronal perikarya in the SNpc is indicated by arrows in f. The optical density of dynein-ir in the striatum ( **g**) and SN ( **h**) was quantified (mean ± SEM) and normalized by the averaged value of nTg group (n = 3). ^***^ *p*<0.001 compared to nTg. A section of the striatum from a 12-month-old homozygous M83 mouse was double labeled with anti-α-synuclein ( **i**, red) and dynein ( **j**, green) antibodies. Note that dynein immunofluorescence intensity was extensively reduced in cells with α-synuclein inclusions (arrows, **i**– **k**). Scale bar = 50 μm in ( **c**– **f**); 20 μm in insets and ( **i**– **k**).

Next, dynein immunohistochemistry was performed on the nigrostriatal sections of the two groups ( Figure 3). An obvious suppression of the dynein immunoreactivity occurred in the striatum in A53T human α-synuclein Tg mice compared with that of nTg mice ( Figure 3c, d). Consistent with the increased protein level of dynein in the ventral midbrain, dynein immunoreactivity in the SNpc was elevated in the transgenic mice ( Figure 3e, f). Abundant dynein accumulation was seen in neuronal perikarya in the SNpc ( Figure 3f). Quantitative analysis of dynein-ir optical density is shown in Figure 3g and h. The data showed that dynein-ir optical density was significantly decreased in the striatum of A53T α-synuclein Tg mice ( *p*<0.001), whereas it was increased in the SNpc ( *p*<0.001).

### Co-localization of dynein and α-synuclein in the striatum of Tg mice

Consistent with a previous report ^13^, we observed A53T α-synuclein inclusions in the striatum ( Figure 3i). Co-localization studies revealed that a marked reduction in dynein immunoreactivity was observed in striatal cells featuring α-synuclein-immunoreactive inclusions compared to cells without α-synuclein inclusions ( Figure 3i–k), suggesting that the reduction in dynein level was associated with accumulation of A53T α-synuclein.

### Relationships between motor behaviors and protein level of dynein

**Figure 4.**
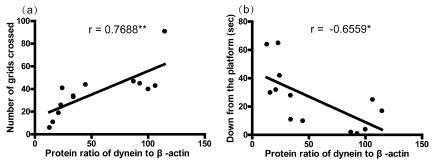
Correlations between the motor behaviors and dynein protein levels in the striatum (n = 13; nTg, n = 5; A53T, n = 8). The correlation analysis between number of grids crossed ( **a**), time to get down from the platform ( **b**), and the protein levels of dynein in the striatum was performed by the Pearson’s correlation test. Asterisks (*) show the significance of the correlation (two tailed). ^*^ *p*<0.05, ^**^ *p*<0.01.

Figure 4 shows a significant positive correlation between the number of total grids crossed and the dynein protein level in the striatum ( Figure 1a, r = 0.7688, *p*<0.01), as well as a negative correlation between the latency to get down from the platform and the dynein protein level in the striatum ( Figure 1b, r = -0.6559, *p*<0.05). These data imply that the dynein protein level in the striatum was correlated with the motor behaviors.

Raw data from behavior tests and dynein protein levels Dataset 1 Raw data of modified open field test.
Two indexes, the time to get down from the platform and the number of grids crossed, are indicated.
Dataset 2 Original western blot images of dynein.
Western blot images of dynein in the midbrain and striatum are shown. In the top panel, the left 3 lanes are from the nTg group and the right 3 lanes are from the A53T group. In the middle panel, the left 2 lanes are from the nTg group and the right 2 lanes are from the A53T group. In the bottom panel, the left 3 lanes are from the nTg group and the right 3 lanes are from the A53T group.
Dataset 3 Quantitation spreadsheet of dynein western blot data.
The ratio of dynein to β-actin was calculated and normalized by the averaged value of nTg group.
Dataset 4 Quantitation spreadsheet of dynein immunohistochemistry data.
The optical density of dynein-ir in the substantia nigra and striatum was quantified and normalized by the averaged value of nTg group.

## Discussion

The major finding of this study is that dynein changes in expression occurred in the nigrostriatal system of A53T human α-synuclein Tg mice, with the level of dynein increasing in the SN, and decreasing in the striatum. At the behavioral level, the alterations were accompanied by significantly reduced horizontal locomotion and prolonged latency in modified open field test ( Figure 1). A correlation analysis showed that the motor behaviors were significantly related to the protein level of dynein in the striatum ( Figure 4). Furthermore, analysis of the co-localization of dynein and α-synuclein in the striatum indicated that the reduction in dynein level was associated with accumulation of A53T α-synuclein ( Figure 3).

The severe motor phenotype was associated with the formation of α-synuclein inclusions in mice expressing A53T human α-synuclein; therefore M83 Tg mice represent an excellent model of α-synucleinopathies (especially familial PD) ^13^. The 10–14 month old Tg mice were too weak to perform in the traditional behavior tests such as the rotarod test and the pole test ^20, 21^, and therefore we used a modified open field test that combines the traditional pole test and open field test. The number of grids crossed was equivalent to the square crossings in the traditional open-field test, a measure of general locomotor activity. The latency to get down from the platform was equivalent to the time to orient downwards in the traditional pole test, which has been used to assess basal ganglia-related movement disorders in mice ^20^. The advantages of this method are the following: (i) the height of the platform is far lower than that of the pole, and, therefore, mice are protected from injuring themselves; (ii) the uneven surface increases the difficulty of movement, thus making the differences in motor function more obvious.

Our data demonstrated that the critical retrograde axonal transport motor dynein was markedly reduced in the striatum of A53T α-synuclein Tg mice, whereas it was upregulated in the SN, indicating defects in retrograde axonal transport in the nigrostriatal pathway. Recent reports have demonstrated that α-synuclein can interact with dynein-containing complexes and its transport involves dynein motor protein ^22^. The reduction in retrograde axonal transport might produce α-synuclein aggregation in neuronal processes (Lewy neurites in the striatum), which provides a reasonable explanation for the fact that aggregation of α-synuclein in neuronal processes was a major feature of M83 Tg mice ^13^. On the other hand, mutant A53T α-synuclein is strongly dependent on autophagy for their clearance ^23^, a dynein-dependent process ^7^. In effect, the abnormalities of dynein would affect the clearance of A53T mutant α-synuclein protein by autophagy. Indeed, our co-localization results suggested that the reduction in dynein level was associated with accumulation of A53T α-synuclein in the striatum ( Figure 3).

Correlation analysis between motor behaviors and dynein demonstrated that the motor behaviors were related to the alteration of dynein protein level in the striatum, which supports the involvement of dynein in neurodegeneration associated with PD and other α-synucleinopathies. In accordance with our findings, a large body of evidence has demonstrated that axonal transport machinery is impaired during neurodegeneration, and likely contributes to this condition ^24, 25^.

Dynein alterations have been detected in several α-synuclein-based models. A report indicated that viral over-expression of human mutant (A53T) α-synuclein resulted in an increase of dynein in striatum but no change in the SN 8 weeks after the injection ^8^. Another report showed a decrease of dynein in the SN 6 weeks following viral A30P α-synuclein over-expression ^9^. These divergent results may be related to the use of different mutant α-synuclein, different promoters, different animal species and different methods of protein evaluation.

This study also raises a few concerns that need to be mentioned. First, the complex changes exhibited by mice expressing A53T human α-synuclein suggest dysfunction in other neuronal systems. The presence of α-synuclein pathology in the motor neurons of the spinal cord may also contribute to motor deficits. Second, although dynein defects occur in the SN, TH-expressing neurons of the SN are spared from pathology. This population of neurons may be protected from the formation of inclusions due to the lack of neuromelanin formation in mice.

## Conclusion

Our results support the idea that dynein changes in the nigrostriatal system of A53T α-synuclein transgenic mice may contribute to their severe movement disorder, which provides new information for understanding the role of dynein in α-synuclein-linked neurodegeneration.

## Data availability

*figshare*: Raw data from behavior tests and dynein protein levels, doi: 10.6084/m9.figshare.954933 ^27^
