# Supplementary figures and images for: Nigrostriatal dynein changes in A53T alpha-synuclein transgenic mice
Source: F1000Res. 2014 Mar 11;3:68. [Version 1] doi: 10.12688/f1000research.3507.1 (PMC4156029; doi:10.12688/f1000research.3507.1)

Dynein  
(Midbrain)

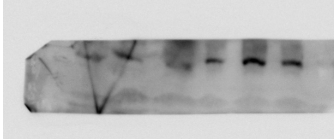

Dynein  
(Midbrain)

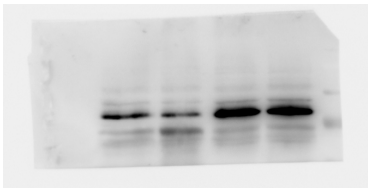

Dynein  
(Striatum)

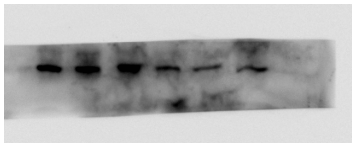

Supplement: Raw data from behavior tests and dynein protein levels — Dataset 1 Raw data of modified open field test. Two indexes, the time to get down from the platform and the number of grids crossed, are indicated. Dataset 2 Original western blot images of dynein. Western blot images of dynein in the midbrain and striatum are shown. In the top panel, the left 3 lanes are from the nTg group and the right 3 lanes are from the A53T group. In the middle panel, the left 2 lanes are from the nTg group and the right 2 lanes are from the A53T group. In the bottom panel, the left 3 lanes are from the nTg group and the right 3 lanes are from the A53T group. Dataset 3 Quantitation spreadsheet of dynein western blot data. The ratio of dynein to β-actin was calculated and normalized by the averaged value of nTg group. Dataset 4 Quantitation spreadsheet of dynein immunohistochemistry data. The optical density of dynein-ir in the substantia nigra and striatum was quantified and normalized by the averaged value of nTg group. [file f1000research-3-3755-s0000.tgz › dataset_2_western_blot_images.pdf]
