# Supplementary material for: Nigrostriatal dynein changes in A53T alpha-synuclein transgenic mice
Source: F1000Res. 2014 Mar 11;3:68. [Version 1] doi: 10.12688/f1000research.3507.1 (PMC4156029; doi:10.12688/f1000research.3507.1)
Supplement: Peer review history

# Peer review: Referee response for version 1

**DOI:** 10.5256/f1000research.3755.r5464

**Published:** 2014-07-28

**Authors:** Abraham Acevedo-Arozena

**Affiliations:** 1 Medical Research Council (MRC) Mammalian Genetics Unit, Harwell Science and Innovation Campus, Oxford, UK

Defects in axonal transport are associated with a number of neurodegenerative disorders, including Parkinson’s disease (PD). Retrograde transport in neurons is powered by the cytoplasmic dynein complex, a macromolecular structure that contains different heavy, intermediate and light dynein polypeptides.  

Here, Liu *et al* report changes in “dynein” protein levels in a widely used mouse model of PD over-expressing mutant (A53T) a-synuclein. Interestingly, the authors found an increase in a-synuclein levels via immunoblot and immunohistochemistry in the substantia nigra (SN) of symptomatic a-synuclein mutant transgenic mice. Conversely, they found a decrease in “dynein” from the striatum of the same mice. The authors used an interesting modification of the open field paradigm to test the mice for motor abnormalities. By comparing the levels of “dynein” in the striatum with the degree of motor deficits in the tested mice, the authors found a correlation between striatum dynein levels and motor performance.

**Comments**:

1. Although the authors refer to “dynein” levels throughout the manuscript, the antibodies they are using for immunoblot are against the cytoplasmic intermediate chain (DIC1?). This needs to be clarified in the materials and also throughout the manuscript.
2. The same applies to the immunohistochemistry. The authors report using two different anti-“dynein” antibodies, but one is against DIC (as above) and the other one against a cytoplasmic dynein light chain (DYNLT3). The authors should clarify which antibody they are using in Figure 3, as well as clarify throughout the text which cytoplasmic dynein subunits they are referring to.  
3. The authors state at the end of the discussion that “ *although dynein defects occur in the SN, TH expressing neurons of the SN are spared from pathology*”. However, they do not show TH co-staining together with a-synuclein in Figure 3. If this statement is referring to previously published data, it should be referenced. If it is referring to their data, it should also appear in Figure 3.
4. In Figure 4, it appears that non-transgenic mice have also been used, together with symptomatic A53T transgenics, for the correlation between the “dynein” levels and the motor abnormalities. I would like to see the same correlation using transgenic mice only, which have obvious motor abnormalities.

I have read this submission. I believe that I have an appropriate level of expertise to confirm that it is of an acceptable scientific standard, however I have significant reservations, as outlined above.

# Peer review: Referee response for version 1

**DOI:** 10.5256/f1000research.3755.r4861

**Published:** 2014-06-05

**Authors:** Gerardo Morfini

**Affiliations:** 1 Department of Anatomy & Cell Biology, University of Illinois at Chicago, Chicago, IL, USA

A large body of pathological evidence has established a “dying-back” pattern on degeneration for neurons affected in Parkinson’s disease (PD). Indeed, axonal processes and synapses of dopaminergic neurons in the substantia nigra (SN) progressively degenerate long before these cells undergo cell death. Mechanisms underlying early degeneration of axons in PD remain unknown, but several lines of evidence have linked this critical pathogenic event to abnormalities in the function of conventional kinesin and cytoplasmic dynein (CDyn), major microtubule-based molecular motors responsible for axonal transport in adult neurons.

Consistent with these observations above, this work by Liu and collaborators reports a striking alteration in the distribution of CDyn in brains of transgenic mice expressing a PD-related mutant form of alpha synuclein (A53T-Tg mice). Immunohistochemical and immunoblotting experiments demonstrated a marked increase in levels of a specific CDyn subunit (DIC) in the SN of A53T Tg mice, compared to age-matched, non-transgenic control mice (nTg mice). Conversely, A53T-Tg mice showed decreased levels of DIC in the striatum when compared to nTg mice. Co-localization studies further suggested that this reduction in striatal DIC levels was associated to accumulation of A53T alpha-synuclein. Additionally, a highly sensitive open-field test paradigm confirmed and further extended prior reports of motor defects in A53T-Tg mice, which significantly correlated with alterations in striatal DIC levels.

Taken together, data from this paper provides novel evidence suggesting that alterations in CDyn function might contribute to disease pathogenesis associated with PD-related mutant alpha synuclein.

**Comments**

 

1. The data presented is consistent with increased activation of CDyn-dependent retrograde axonal transport by mutant alpha synuclein. Supporting this view, prior studies showed activation of CDyn-based retrograde transport by the parkinsonian drug MPP+ ( Kim-Han JS *et al*., *J Neurosci.* 2011). However, this possibility is not considered in the Discussion section.
2.  CDyn is composed of multiple protein subunits including heavy (DHC) intermediate (DIC), and light (DLC) subunits, among others. In Materials and Methods, the authors report using both a monoclonal (clone 74.1; Millipore#MAB1618), and a polyclonal (Abcam ab81507) antibody against DICs, but is unclear which one was used for immunoblots shown in Figure 2. Throughout the manuscript and in the Figure 2 legend, the text should clearly indicate that data gathered in Figure 2 corresponds to DIC subunits, rather than “dynein”.
3.  The authors report using an antibody from Millipore (presumably #MAB1618 against DIC), and a polyclonal (Abcam ab121209) antibody against TcTtex (one of several DLC subunits). Again, the main text and Figure 3 legend should indicate which specific CDyn subunits are analyzed for each panel in Figure 3.
4.  Some type of quantitation could be provided to illustrate the negative correlation between TcTtex levels and A53T alpha-synuclein accumulation.

 

**Minor comments**

 

1. The authors mention that the major finding in their study is that changes in CDyn expression occurred in the nigrostriatal system of A53T human α-synuclein Tg mice. However, the experiments performed do not directly address protein expression. Rather, those evaluate overall DIC steady state levels.
2.  To ensure reproducibility of results by other investigators, please provide the lot number for Abcam polyclonal antibodies #ab81507 and #ab121209.

I have read this submission. I believe that I have an appropriate level of expertise to confirm that it is of an acceptable scientific standard.

# Peer review: Referee response for version 1

**DOI:** 10.5256/f1000research.3755.r4454

**Published:** 2014-05-06

**Authors:** James B Koprich

**Affiliations:** 1 Toronto Western Research Institute, Toronto Western Hospital, University Health Network, Toronto, ON, Canada

**Summary**

In this paper by Liu *et al.* a relationship between alpha-synuclein accumulation, motor deficits and expression of dynein in the nigrostriatal pathway is described. The study used transgenic mice overexpressing A53T aSyn similar to the M83 line and age matched Wt littermate controls. A behavioural deficit was confirmed in the Tg mice using a modified open-field test. The Tg mice showed reduced exploration and increased latency to get down from the starting platform. Mice were then sacrificed and tissue was prepared for western blot of dynein and immunohistochemistry of dynein and aSyn. The results showed decreased levels of dynein in the striatum and increased levels in the SN, both from western blots and histology. Furthermore, the authors showed that dynein levels were reduced in neurons with higher level of aSyn.

**Comments**

1. The number of animals used in each analysis needs to be provided along with the degrees of freedom for each statistical test performed.
2. The staining in Fig 3, panel F has much more background compared to its counterpart in panel E. Please confirm that cases from different groups were all stained together to rule out any histology related confounds.
3. The results from Fig 3 panels I, J and K are taken from a single region of a single case. Random sampling within the region of interest should be performed on each case (compared to Wt controls) to arrive at any conclusion. Optical density or fluorescent intensity measures should be obtained in the process to provide a quantitative assessment.
4. The co-staining in Fig 3, panel K should include TH to know that the neurons being analyzed are SN dopamine neurons as alluded to in the conclusion.  

I have read this submission. I believe that I have an appropriate level of expertise to confirm that it is of an acceptable scientific standard, however I have significant reservations, as outlined above.
